# N-acetylcysteine Clinical Applications

**DOI:** 10.7759/cureus.72252

**Published:** 2024-10-24

**Authors:** Zoubaida Yahia, Amer Yahia, Tarek Abdelaziz

**Affiliations:** 1 General Practice, Wrightington, Wigan and Leigh NHS Foundation Trust, Wigan, GBR; 2 Medical Assessment Unit, Royal Derby Hospital, Derby, GBR; 3 Acute Medicine, Blackpool Victoria Hospital, Blackpool, GBR

**Keywords:** adjunct therapy, cataracts, chronic diseases, endometriosis, glaucoma, male infertility, n-acetylcysteine, neurodegenerative diseases, polycystic ovary syndrome, psychiatric disorders

## Abstract

This study aims to evaluate the therapeutic application of N-acetylcysteine (NAC) as a treatment or adjunct therapy for various medical conditions. While its efficacy in treating acetaminophen overdose, cystic fibrosis, and chronic obstructive pulmonary disease is well-established, emerging evidence suggests that NAC may also benefit a broader spectrum of illnesses due to its safety, simplicity, and affordability. A comprehensive review was conducted by searching PubMed, relevant books, and conference proceedings for publications discussing NAC about the specified health conditions. The clinically relevant data were analysed using the American Family Physician Evidence-Based Medicine Toolkit, following a standard integrated review methodology. NAC shows potential as an adjunctive treatment for a wide range of medical conditions, particularly chronic diseases. It may be beneficial for polycystic ovary syndrome, endometriosis, male infertility, cataracts, glaucoma, dry eye syndrome, parkinsonism, multiple sclerosis, Alzheimer’s disease, stroke outcomes, non-acetaminophen-induced acute liver failure, Crohn’s disease, ulcerative colitis, schizophrenia, bipolar disorder, and obsessive-compulsive disorder. Although evidence for some conditions is less robust, NAC's therapeutic potential warrants further investigation. Given the aging population and the decline in glutathione levels, the use of NAC should be considered across a variety of medical conditions. This paper suggests that NAC supplementation could play a significant role in reducing morbidity and mortality associated with numerous chronic diseases.

## Introduction and background

N-acetylcysteine (NAC), a sulfhydryl molecule, is an amino acid derivative of L-cysteine. Since 1969, NAC's mucolytic action has been employed in the treatment of cystic fibrosis for the first time [1]. It was soon utilised as an antidote in the treatment of acetaminophen intoxication [2]. It is now recognised primarily as an antioxidant with direct and indirect actions [3]. Oxidative stress is involved in the pathogenesis of many diseases, including cancer, cardiovascular disease, arthritis, diabetes, influenza-like symptoms, and some lung disorders such as pulmonary oxygen toxicity, adult respiratory distress syndrome, chronic obstructive pulmonary disease (COPD), idiopathic pulmonary fibrosis [4], and cystic fibrosis. An increasing number of articles [3,5] confirm the efficacy of NAC use in the aforementioned disorders. Natural sources of cysteine include meat, fish, cereals, dairy, soybean, and egg products [6]. NAC is available in modest levels naturally in several fruits and vegetables as a dietary supplement [7].

## Review

Methods

A comprehensive review of the literature was conducted to evaluate the clinical applications and therapeutic significance of NAC. Key databases, including PubMed and MEDLINE, were searched using relevant keywords such as "N-acetylcysteine", "adjunct therapy", and "chronic diseases". The search focused on human studies published over the last four decades, prioritising randomised controlled trials (RCTs) and meta-analyses to ensure strong clinical evidence. Preclinical studies involving cell cultures and animal models were included when applicable to support mechanistic insights into NAC's effects.

To assess the quality and strength of evidence supporting NAC's clinical use in various contexts (e.g., acetaminophen toxicity, chronic obstructive pulmonary disease), the American Family Physician (AFP) Evidence-Based Medicine Toolkit was employed [5]. This toolkit facilitated the categorisation of clinical recommendations according to their evidence strength, ensuring that all therapeutic applications discussed in this review are grounded in a clear, evidence-based rationale. A PubMed search yielded 25,310 publications, with an additional 80 articles obtained via Medline. After reviewing 25,390 items, 25,021 were removed. Following a full-text eligibility evaluation, 369 articles were assessed. Of these, 78 were included, whereas 291 were eliminated for the following reasons: 73 were out of scope, 139 were repeats of previously included material, 13 lacked appropriate information, and 66 had irrelevant results.

Mechanism of Actions

NAC has emerged as a prominent antioxidant compound in both experimental and clinical research settings. Its widespread adoption in cellular, animal, and human studies has established it as an antioxidant. While numerous investigations have employed NAC to mitigate oxidative stress, the molecular mechanisms underlying its therapeutic effects warrant careful consideration [8].

**Figure 1:**
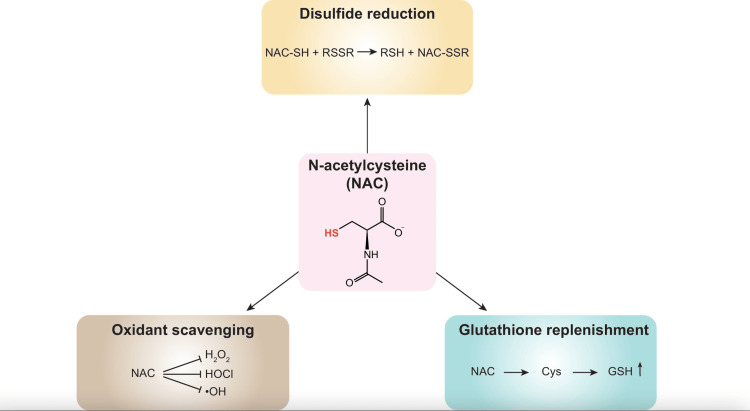
Graph demonstrating the application N-acetylcysteine (NAC)'s biological effects. Source: [8]

Three principal mechanisms have been proposed to explain NAC's biological activities, as shown in Figure 1 [8]. The first one is a disulphide reductant, which modulates extra- and intracellular redox states through a direct reduction of disulfide bonds. The second one suggests direct scavenging of reactive oxygen species, including hydrogen peroxide (H_2_O_2_), hypochlorous acid (HOCl), and hydroxyl radicals (•OH). The third one proposes that NAC a cysteine donor, which enhances cellular glutathione synthesis and thereby augments antioxidant capacity. However, these proposed mechanisms, while potentially valid in specific contexts, may not fully account for NAC's therapeutic effects across all clinical applications, as the mechanism of action is not fully tested in some cases.

Indications

Eye Disorders

The function of oxidative processes in diabetes, age-related macular degeneration (AMD), dry eye syndrome, and cataracts appear to be critical for the progression of pathological alterations in ocular tissues. The eye is extremely vulnerable to oxidative stress. Molecular oxygen can directly destroy or cause secondary reactions that might launch oxidative activities [6].

AMD: The addition of NAC to cell cultures of retinal pigment epithelium resulted in a significant reduction of oxidative damage in cell studies [7]. NAC also upregulates reduced glutathione production and also reverses lipid peroxidation in these cells [9]. This has been suggested as a new treatment for macular degeneration and clinical studies are warranted.

Dry eye syndrome: Acetylcysteine eye drops are an excellent treatment option for those who have sticky, viscous mucus on their eyes (filamentary keratitis). NAC (200 mg three times per day) reduced eye-related symptoms in a double-blind, placebo-controlled crossover experiment of 26 individuals with Sjögren's disease. 

It has also shown some potential for mouth-related problems, although the results were mixed [10]. Recent clinical trials [11] suggest that topical NAC treatment is extremely beneficial in individuals with meibomian gland dysfunction. There was a significant improvement in the symptoms of itching and burning, as well as an increase in the average of the Schirmer tear test.

Cataracts: Because glutathione is an essential antioxidant in the lens, boosting GSH levels with NAC supplementation may be utilised to prevent cataract risk [12]. Jain et al. [13] investigated the pathophysiology of early cataract formation and the possible effect of vitamin B6 and NAC supplementation in the diabetic population. High glucose concentrations can promote protein oxidation and alteration in the lens. Supplementing with vitamin B6 (pyridoxine) and NAC may help decrease the oxidation of lens proteins. Liebermann's work [14] suggested the idea that NAC treatment may correct early cataracts.

Glaucoma: Currently, animal studies show that NAC may reduce retinal damage induced by ocular hypertension [15]. NAC, which raises glutathione, inhibited oxidative stress and autophagy, suggesting that it may be effective in some types of glaucoma patients [16].

Neurological Disorders

NAC appears to be protective against neurodegenerative illnesses such as Parkinson's disease, Alzheimer's disease, stroke, and multiple sclerosis (MS). It may be useful as an adjuvant for these illnesses since it is a glutathione precursor with antioxidant and anti-inflammatory effects [17,18].

Alzheimer's disease: NAC has been investigated in certain mouse models of Alzheimer's disease, and these investigations showed evidence that NAC treatment reduces oxidative damage in Alzheimer's disease [19,20]. Adair et al. [21] used NAC in a blinded placebo-controlled experiment in individuals with Alzheimer's disease. NAC therapy did not affect the key outcome indicators in people with clinically confirmed Alzheimer's disease. However, the findings may promote further research into NAC in Alzheimer's disease.

Parkinson’s disease: Dopamine may cause apoptosis in neuronal cell cultures, resulting in the loss of nigral cells in Parkinson's disease. Thiol-containing drugs, such as NAC, are very protective in cell cultures by reducing dopamine-induced cell death [22]. Over three months, a clinical study employing NAC as a weekly intravenous infusion and 500 mg orally twice a day dramatically reduced Parkinson's symptoms and enhanced dopamine binding in the brain, warranting additional research [23].

Multiple sclerosis: NAC reduces tumour necrosis factor toxicity, and in an animal model of multiple sclerosis, it prevents the development of multiple sclerosis-like pathology [24]. Ten multiple sclerosis patients were given NAC treatment for up to 16 months. Due to the relapsing-remitting nature of many MS patients, determining the effectiveness of NAC in a small sample without contemporaneous controls is problematic. However, two MS patients with a lengthy history of difficulties speaking had a very substantial improvement in their speech immediately after beginning to use NAC. Controlled studies are required to determine whether NAC can reduce the amount of MS exacerbations [25].

Stroke: Acrolein-mediated damage after stroke has been linked to stroke size in animal studies, and NAC has been demonstrated to diminish infarct size [26]. A recent randomised double-blind placebo-controlled trial employing NAC 4 grams four times a day for 72 hours in the beginning (within 24 hours) of an ischemic stroke resulted in an improved outcome profile in both neurological impairment and disability at 90 days [27].

Psychological Disorders

NAC is being investigated as an additional treatment for a variety of psychiatric illnesses. The use of NAC as an additional therapy to lower the overall and negative symptoms of schizophrenia is supported by strong evidence. NAC appears to be useful in lowering craving in drug use disorders, particularly in the treatment of cocaine and cannabis use among young people, as well as in avoiding relapse in persons who are already abstinent. Even though encouraging data exist, the effects of NAC on obsessive-compulsive and associated disorders, as well as mood disorders, remain uncertain, with conflicting reviews [28].

Addiction and substance use disorders: NAC has been proven in preclinical studies to be capable of restoring the imbalance of cysteine-glutamate exchange in the brain and decreasing drug-seeking behaviours in animal models [29]. Indeed, NAC therapy for addiction and drug abuse problems has been a focus of recent studies. Many clinical trials have been undertaken in the recent decade, and their findings have been summarised in five systematic reviews published in the previous four years. Nocito Echevarria et al. [30] conducted a thorough assessment of animal research and clinical trials on NAC therapy for cocaine dependency. Based on the outcomes of four clinical studies, NAC was found to lower craving, desire to use cocaine, cocaine-cue viewing time, and cocaine-related expenditure [30]. According to animal research, the favourable impact was perhaps attributable to the restoration of glutamate equilibrium. Nonetheless, NAC did not affect abstinence in a major double-blind placebo-controlled experiment with 111 cocaine-addicted treatment-seeking individuals [31]. As a result, the authors hypothesised that NAC would be more suited for relapse prevention in those who are already abstinent [30].

Schizophrenia: NAC operates at many sites throughout the brain to enhance anti-schizophrenia activity. Modulating neuroinflammation related to cell malfunction and death, encouraging neurogenesis and neuronal injury repair, and normalising glutamate dysregulation such as N-methyl-D-aspartate hypofunction are among these activities [32,33,34,35]. In one clinical experiment, patients treated with NAC had higher multivariate phase synchronisation, which changed neuron connectivity in the brain, as evaluated by an electroencephalogram, even before any clinically noticeable improvement [36]. Only one systematic review of clinical trials of NAC in schizophrenia was discovered in the literature. Chen et al. [37] included just two double-blind, placebo-controlled studies and discovered that supplementary NAC may be effective in lowering negative and general schizophrenia symptoms. Zheng et al. [38] found that NAC substantially reduced overall symptom ratings in schizophrenia in three RCTs involving 307 subjects (NAC: 153, placebo:154).

Obsessive-compulsive and related disorders: Patients with obsessive-compulsive disorder have higher levels of glutamate in their cerebrospinal fluid [39]. Because of its capacity to decrease synaptic glutamate release via the glial cysteine-glutamate exchange, NAC has been proposed as a therapeutic therapy for this disease [39]. In their systematic review, Minarini et al. [40] included nine clinical studies on the treatment benefits of NAC on obsessive-compulsive and associated disorders (obsessive-compulsive disorder: 3, Tourette syndrome: 1, trichotillomania: 2, excoriation: 2, and onychophagia: 1) Based on the outcomes of these nine clinical studies and 11 published case reports/series, the authors concluded that the findings are preliminary. Excoriation appears to be the most potential region for NAC use among these disorders [40].

Mood disorders: Fernandes et al. [41] conducted a comprehensive review and meta-analysis of double-blind, placebo-controlled trials of NAC for depressive symptoms independent of the underlying mental disease. NAC was proven to significantly alleviate depression symptoms and enhance functioning compared to placebo in a pooled analysis of data from 574 people (NAC: 291, placebo: 283) [41]. There was insufficient evidence to accurately analyse the effects of NAC on the quality of life and manic symptoms, according to the authors [41]. Treatment-resistant subthreshold depression is a key concern in bipolar illness. An RCT employing NAC as an augmentation method for bipolar disorder depressed symptoms was reported to be safe and effective [42].

Pulmonary Diseases

Research on pathological lung problems is a growing field, with early beginnings employing inhaled mucomyst for cystic fibrosis. Oral NAC is increasingly being utilised to treat illnesses such as chronic obstructive pulmonary disease and the disorders listed below [43,44].

Chronic obstructive pulmonary disease: After two months of therapy, open-label research of 1,392 patients discovered that NAC reduced the viscosity of expectorated phlegm, reduced cough intensity, and increased ease of expectoration in 80%, 74%, and 71% of patients, respectively [43]. The research also found "significant improvements" in rhonchi, crepitations, dyspnoea, cyanosis, and associated heart failure after one to two months of medication [43]. In another large, open-label experiment that compared NAC to a controlled drug, patients using NAC had less deterioration of lung function as determined by forced expiratory volume in one second (FEV) [44]. NAC has also been investigated for its ability to reduce COPD exacerbations. A meta-analysis of 11 double-blind, placebo-controlled trials chosen from 39 available studies based on quality criteria demonstrated a statistically significant difference in the number of exacerbations in patients treated with NAC versus those receiving placebo [45].

Cystic fibrosis: NAC was hypothesized to have a beneficial therapeutic impact on CF patients as a mucoactive, anti-inflammatory, and antioxidant drug. NAC was given orally to 18 CF patients at high dosages (600-1000 mg) three times daily during a four-week phase I study [46]. Elastase activity, neutrophil load, and IL-8 levels in airway fluid collected by sputum induction decreased considerably. Furthermore, NAC treatment resulted in the restoration of circulating neutrophils and GSH shortage, as well as a decrease in the number of airway neutrophils. A phase II randomized, placebo-controlled clinical study [47] examined the influence of low (700 mg) or high (2800 mg) doses of NAC in a 12-week treatment of 21 patients with cystic fibrosis. High NAC doses were well tolerated but had no effect on clinical or inflammatory parameters except extracellular glutathione level, which slightly increased. Further studies are needed to learn if NAC can indeed be useful in cystic fibrosis therapy.

Asthma and allergy: Currently, there is no evidence to support the use of NAC in acute asthma episodes due to the absence of improvement in cough, wheezing, dyspnoea, sputum expectoration, or night sleep [48]. NAC did, however, improve steroid-resistant acute asthma exacerbations in animal models [49]. There have been no long-term trials that have used NAC to prevent repeated asthma episodes by lowering inflammation and mucus plugging. In animal models, NAC has been demonstrated to decrease the allergen-induced nasal inflammatory cascade in allergic rhinitis [50]. Topical NAC use before ragweed exposure reduced late-phase allergy reaction-mediated nasal symptoms [51].

Idiopathic pulmonary fibrosis: IFIGENIA randomized placebo-controlled one-year studies with high dosages (1800 mg/day) of NAC added to azathioprine and prednisone therapy were done in 182 patients. Disease development was greatly halted as evaluated by vital capacity and diffusing capacity [52,53]. Bennet [54] produced similar results in a small randomised study using high NAC dosages in addition to the usual treatment. Other studies, on the other hand, have not shown NAC to be helpful [55, 56].

Liver and Bowel Diseases

Liver and bowel illnesses are quite common around the world. The most prominent pathogenetic processes in liver and bowel illnesses are oxidative stress (OS) and inflammation, which are recognized to play a vital role in several pathologies such as acetaminophen overdose, ulcerative colitis, Crohn's disease, and non-acetaminophen-induced acute liver failure [57].

Acetaminophen overdose: The effectiveness of NAC in preventing acute liver failure has been well demonstrated when administered intravenously within eight hours after consumption [57]. NAC was originally mentioned as an antidote for acetaminophen-induced (AI) liver damage in 1977 and became more commonly acknowledged by the mid-1980s [58]. Glutathione detoxifies the reactive metabolite of acetaminophen. NAC replenishes glutathione levels, avoiding irreparable damage [58].

Non-acetaminophen-induced acute liver failure: NAC has been used to treat mushroom poisoning [59], pesticide (Paraquat) poisoning [60], chloroform poisoning [61], polychlorinated biphenyls (PCB)-induced steatosis [62], and other poisonings. NAC has been utilised to treat acetaminophen damage because of its antioxidant, anti-inflammatory, and vasodilating properties [63]. The administration of NAC in NAI-ALF decreased mortality, duration of stay, and enhanced survival [64,65]. A meta-analysis of prospective clinical studies comparing NAC-treated and placebo-treated groups found that NAC was safe and extended the lives of patients with native livers who did not require transplantation, but it did not increase overall survival [66].

Ulcerative colitis: The use of antioxidant treatment in inflammatory bowel disease has already been proposed [67]. The recurrence rate in an RCT utilizing 400 mg of NAC twice a day in ulcerative colitis patients on prednisolone taper was considerably lower in the therapy group. The therapy group had a decreased endoscopic recurrence rate, serum level of high-sensitivity C-reactive protein (hs-CRP), and faecal calprotectin level [68].

Crohn’s disease: Even in clinical remission, Crohn's disease causes significant systemic oxidative stress [69]. In a double-blind RCT, individuals using NAC 400 mg twice daily had a lower relapse rate than those taking a placebo while decreasing prednisolone [70].

Gynaecological Disorders

Because of its impact on hyperinsulinemia, NAC has shown significant success not just in treating women with polycystic ovarian syndrome (PCOS) but also in treating endometriosis by lowering oxidative stress.

Endometriosis: NGO [71] investigated the impact of NAC on endometriotic cells taken from endometriosis patients and a mouse model of endometriosis. They discovered a link between increased ROS generation, altered ROS detoxification routes, decreased catalase levels in endometriotic cells, cellular proliferation, and ERK1/2 MAP kinase activation. Proinflammatory cytokines and growth factors, as well as indicators of oxidative stress, were shown to be higher in endometriosis patients' peritoneal fluid [71,72,73]. The cells were treated with increasing concentrations of NAC, with the dosage determined by the suppression of intracellular H_2_O_2 _concentration and proliferation rate. The RAF/MEK/ERK signalling pathway was activated in endometrial and stromal cells from patients with endometriosis, as well as in endometriotic cells. After two hours of incubation with NAC at a concentration of 10 mmol/l, the pERK level was reduced to that of control cells (stromal and epithelial cells from healthy women). NAC appears to be promising in endometriosis treatment since it can lower oxidative stress, chronic inflammation, and high angiogenesis levels [74,75].

PCOS: NAC has a favourable effect on PCOS. This antioxidant was supplied to hyperinsulinemic PCOS women as an additional treatment to clomiphene and improved their insulin sensitivity and circulating insulin level [76]. Masha et al.'s study [77] produced a similar outcome. NAC (1200 mg/day) and nitric oxide precursor/L-arginine (1600 mg/day) treatment improved insulin sensitivity and restored gonadal function in PCOS women. Other studies found that NAC used in clomiphene citrate (CC)-resistant women with PCOS increased ovulation and conception by a clinically and statistically meaningful amount [78,79]. In women with POCS, NAC at a dosage of 1200-1800 mg/day appears to be beneficial, while metformin treatment appears to be more successful [79].

## Conclusions

When taken as a supplement or in the treatment of different illnesses, NAC appears to be well tolerated with few adverse effects. As previously indicated, the dose for this prescription is not always evident, and significant work is necessary to supply this information. These acts explain some of the outcomes observed in a wide range of settings. As our understanding of NAC increases, many new circumstances emerge that are not covered in this paper. As with other antioxidants in the past, there is some caution with lung cancer models, where there may be an increase in proliferation as a result of p53 suppression, as well as an increase in pulmonary hypertension.

As previously stated, the benefit has been demonstrated in the treatment of pulmonary, mental, neurologic, metabolic, and viral disorders, reproductive difficulties, and certain malignancies. NAC can be used as an adjuvant for the majority of these disorders, potentially improving quality of life, morbidity, and mortality.

However, despite its great promise, NAC's clinical effectiveness has limits. One notable disadvantage is that dose and duration vary throughout research, making it difficult to standardise therapy regimens. The diversity in patient response complicates its therapeutic uses, since illness stage, comorbid diseases, and individual biochemistry can all impact results. While NAC has shown promise in neurodegenerative illnesses such as Parkinson's and Alzheimer's, human trials have had conflicting results, with some research indicating minor therapeutic effects. Similarly, some studies have supported its usefulness in respiratory disorders such as COPD and cystic fibrosis, while others have questioned it owing to a lack of long-term evidence and small sample numbers.

Although preliminary data from animal research provide light on its processes, more rigorous clinical evidence is required to thoroughly assess its therapeutic potential, particularly in illnesses such as endometriosis, ulcerative colitis, and addiction problems. The use of animal models and small human cohorts restricts the generalisability of these findings, emphasising the need for more studies with bigger, more varied populations.
